# Usenamine A triggers NLRP3/caspase-1/GSDMD-mediated pyroptosis in lung adenocarcinoma by targeting the DDX3X/SQSTM1 axis

**DOI:** 10.18632/aging.205450

**Published:** 2024-01-23

**Authors:** Min Li, Rongrong Wu, Le Wang, Dongyi Zhu, Shinan Liu, Ruolan Wang, Chaowen Deng, Shenglin Zhang, Min Chen, Ruojin Lu, Hongxing Zhu, Mengting Mo, Xiaoqiong He, Zhuang Luo

**Affiliations:** 1 Department of Respiratory and Critical Care Medicine, The First Affiliated Hospital of Kunming Medical University, Kunming 650032, China; 2 Department of Radiology, The First People’s Hospital of Yunnan Province (Affiliated Hospital of Kunming University of Science and Technology), Kunming 650034, China; 3 School of Public Health, Kunming Medical University, Kunming 650500, China

**Keywords:** usenamine A, lung adenocarcinoma, pyroptosis, NLRP3/caspase-1/GSDMD, DDX3X

## Abstract

Background: Usenamine A (C_18_H_17_NO_6_) is a newly developed, natural anticancer drug that reportedly exerts low toxicity. The therapeutic efficacy and underlying mechanisms of usenamine A in lung adenocarcinoma (LUAD) remain poorly understood. We aimed to explore the therapeutic effects and molecular mechanisms through which usenamine A inhibits LUAD tumorigenesis.

Methods: We used LUAD cell lines H1299 and A549 in the present study. CCK-8 and colony formation assays were performed to analyze cell proliferation. Cell migration, invasion, and apoptosis were evaluated using wound-healing, transwell, and flow cytometric assays, respectively. Levels of reactive oxygen species were measured using a DCFH-DA probe. Inflammatory factors (lactate dehydrogenase, interleukin [IL]-1β, and IL-18) were detected using enzyme-linked immunosorbent assays. Western blotting was performed to determine the expression of NOD-like receptor pyrin 3 (NLRP3)/caspase-1/gasdermin D (GSDMD) pathway-related proteins. Pyroptosis was detected using transmission electron microscopy. The interaction and co-localization of DDX3X and sequestosome 1 (SQSTM1) were identified using co-immunoprecipitation and immunofluorescence assays, respectively. For *in vivo* assessment, we established a xenograft model to validate the usenamine A-mediated effects and mechanisms of action in LUAD.

Results: Usenamine A inhibited the proliferation, migration, and invasion of LUAD cells. Furthermore, usenamine A induced NLRP3/caspase-1/GSDMD-mediated pyroptosis in LUAD cells. Usenamine A upregulated DDX3X expression to trigger pyroptosis. DDX3X interacted with SQSTM1, which is responsible for inducing pyroptosis. *In vivo*, usenamine A suppressed LUAD tumorigenesis by triggering NLRP3/caspase-1/GSDMD-mediated pyroptosis via the upregulation of the DDX3X/SQSTM1 axis.

Conclusions: Usenamine A was found to induce NLRP3/caspase-1/GSDMD-mediated pyroptosis in LUAD by upregulating the DDX3X/SQSTM1 axis.

## INTRODUCTION

Lung adenocarcinoma (LUAD) is the most common subtype of lung cancer, known for its high mortality rate [1, 2]. It is predicted that lung cancer will be responsible for 609,360 deaths in 2022 in the United States, equating to approximately 350 deaths daily [3]. LUAD accounts for approximately 50% of all lung cancer cases worldwide, with a 5-year survival rate at only 22% [3, 4]. Currently, radiation and chemotherapy are first-line therapies for LUAD; however, these treatment strategies may cause severe adverse effects and afford a poor prognosis [5]. Other therapeutic interventions, including lobectomy and segmentectomy, are mainly effective for early-stage LUAD [6]. Therefore, it is crucial to explore effective adjuvant drugs and elucidate their mechanisms of action, contributing to developing novel therapeutic strategies for LUAD.

Pyroptosis is a form of proinflammatory programmed cell death that is increasingly recognized [7, 8]. Pyroptosis is mediated by caspase-gasdermin D (GSDMD) signaling pathways, which is activated by NOD-like receptor pyrin 3 (NLRP3) inflammasome [9]. NLRP3/caspase-1/GSDMD-mediated pyroptosis is emerging as a promising therapeutic target in cancer treatment, thereby leading to the search for effective drugs and biomarkers for cancer treatment. A previous study revealed that circNEIL3 can regulate NLRP3/caspase-1/GSDMD-mediated pyroptosis to influence radiotherapy targeting LUAD [8], demonstrating the pivotal role of pyroptosis in LUAD. Usenamine A (C_18_H_17_NO_6_), a compound derived from the Usnea plant in Yunnan (China), is a novel natural anticancer drug with low toxicity both *in vitro* and *in vivo* (Patent No.:201710388136.8). *In vitro* experiments have revealed that usenamine A can exert a notable inhibitory effect on lung, bladder, and liver cancers, and was found to be superior to cisplatin and paclitaxel. He et al. confirmed that usenamine A combined with scutellarin promotes apoptosis of human glioma cells and impairs glioma cell survival [10, 11]. However, the therapeutic effects of usenamine A on LUAD remain poorly explored. This study investigates whether usenamine A regulates NLRP3/caspase-1/GSDMD-mediated pyroptosis in LUAD.

DEAD-Box helicase 3 X-Linked (DDX3X), a stress granule protein, interacts with NLRP3 to influence the assembly of NLRP3 inflammasome [12]. The initiation of pyroptosis via the NLRP3/caspase-1/GSDMD pathway critically depends on the involvement of DDX3X [12]. Given its central role in inflammasome-mediated cell death, and considering the frequent mutations of DDX3X found in various cancers, it presents as a potential target for anticancer therapies [13, 14]. Wu et al. showed that the loss of DDX3 can facilitate malignant tumor progression and indicates a poor prognosis of non-small cell lung cancer [15]. In addition, DDX3X has been shown to promote sequestosome 1 (SQSTM1/p62) accumulation in pancreatic ductal adenocarcinoma [16], suggesting a regulatory relationship between DDX3X and SQSTM1. SQSTM1 is a critical autophagy receptor that regulates inflammatory response [17]. Cao et al. showed that SQSTM1 accumulation could exacerbate NLRP3-independent cell death via GSDMD-mediated pyroptosis [18]. Moreover, elevated SQSTM1 expression was closely correlated with poor prognosis of LUAD [19]. The specific mechanism of the DDX3X/SQSTM1 axis in NLRP3/caspase-1/GSDMD-mediated pyroptosis in LUAD remains largely unknown.

Herein, we hypothesized that usenamine A induces NLRP3/caspase-1/GSDMD-mediated pyroptosis in LUAD by targeting the DDX3X/SQSTM1 axis. Accordingly, we examined the function of usenamine A in LUAD considering the NLRP3/caspase-1/GSDMD-mediated pyroptosis and determined the mechanism involved in the DDX3X/SQSTM1 axis. This exploration could pave the way for novel therapeutic strategies in the treatment of LUAD.

## RESULTS

### Usenamine A induces NLRP3/caspase-1/GSDMD-mediated pyroptosis in LUAD cells

**Figure 1:**
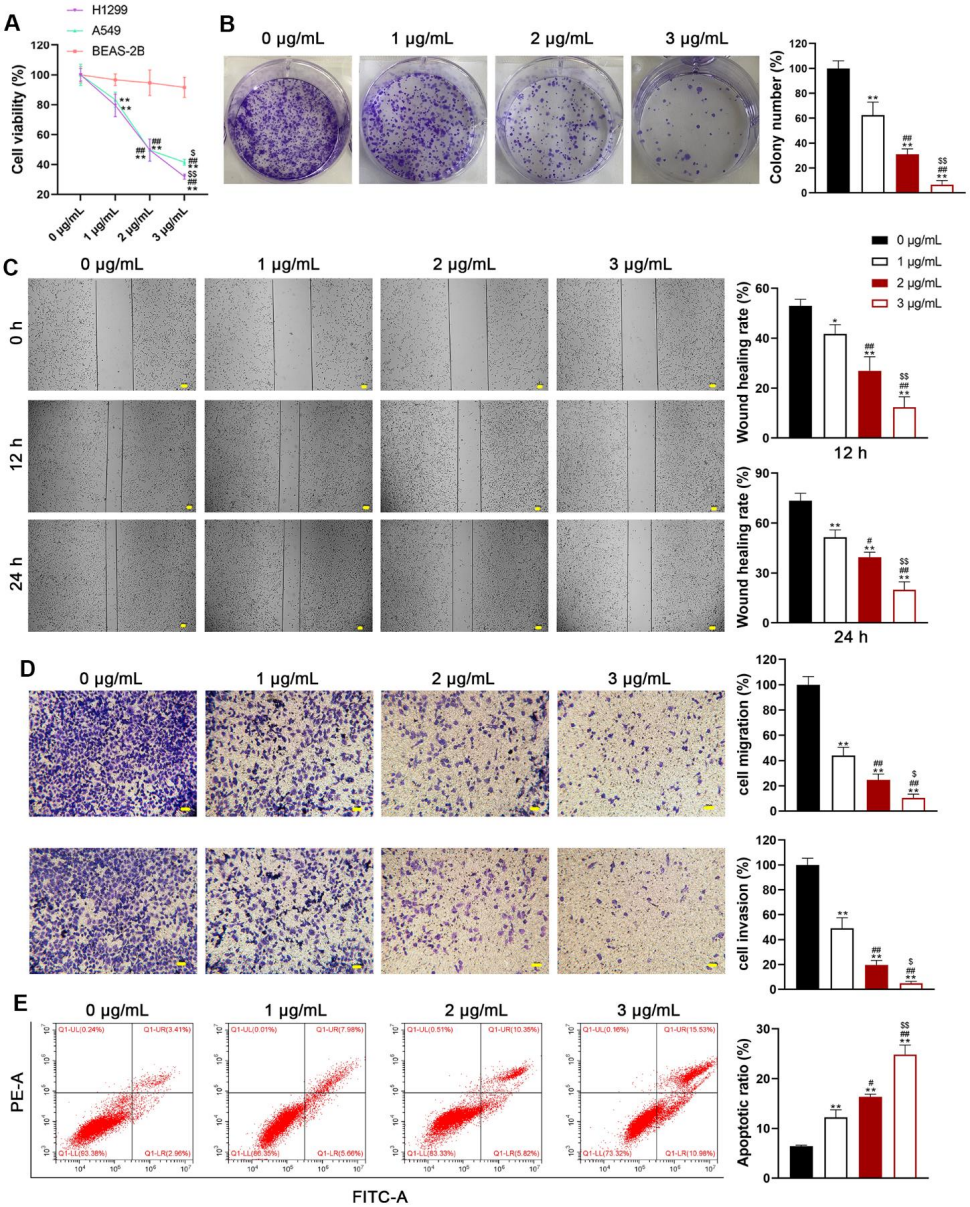
**Usenamine A inhibits the proliferation, migration, and invasion of LUAD cells.** (**A**) The viability of human LUAD cell lines (H1299 and A549) and normal lung epithelial cells (BEAS-2B) was measured using the CCK-8 assay. (**B**) H1299 cell proliferation was detected using the colony formation assay. (**C**) H1299 cell migration was measured using the wound-healing assay. Scale bar = 50 μm. (**D**) H1299 cell migration and invasion were determined using the transwell assay. Scale bar = 50 μm. (**E**) H1299 cell apoptosis was assessed by flow cytometry. *p < 0.05 and **p < 0.01 vs*.* 0 μg/mL usenamine A; #*p* < 0.05 and ##*p* < 0.01 vs*.* 1 μg/mL usenamine A; $*p* < 0.05 and $$*p* < 0.01 vs*.* 2 μg/mL usenamine A.

**Figure 2:**
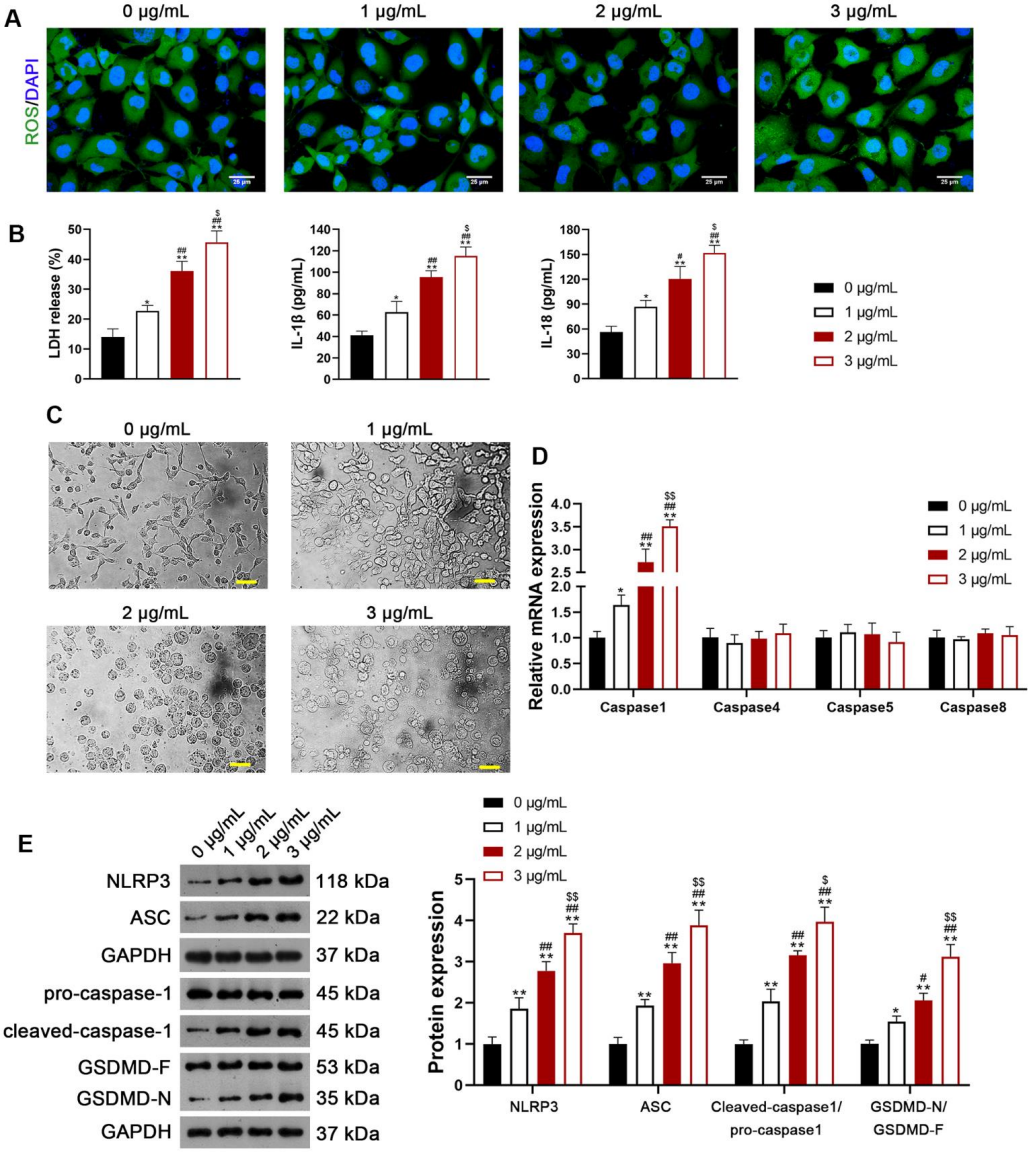
**Usenamine A induces NLRP3/caspase-1/GSDMD-mediated pyroptosis in LUAD cells (H1299).** (**A**) ROS levels in the cells were detected using a DCFH-DA probe. Scale bar = 25 μm. (**B**) LDH, IL-1β, and IL-18 levels in the cells were measured using commercial ELISA kits. (**C**) Representative morphological images of cells obtained by microscopic examination. Scale bar = 50 μm. (**D**) Relative expression of caspase-1, -4, -5, and -8 was analyzed using RT-qPCR. (**E**) Relative protein expression of NLRP3/caspase-1/GSDMD pathway-related proteins was measured using western blotting. *p < 0.05 and **p < 0.01 vs*.* 0 μg/mL usenamine A; #*p* < 0.05 and ##*p* < 0.01 vs*.* 1 μg/mL usenamine A; $*p* < 0.05 and $$*p* < 0.01 vs*.* 2 μg/mL usenamine A. GSDMD, gasdermin D; LUAD, lung adenocarcinoma; NLRP3, NOD-like receptor pyrin 3; RT-qPCR, reverse transcription-quantitative PCR.

We selected H1299 and A549 LUAD cell lines, representing p53-null and p53-functional statuses, to assess the differential impact of usenamine A (0, 1, 2, and 3 μg/mL). Cell counting kit (CCK)-8 assay revealed that usenamine A dose-dependently inhibited cell viability, and the IC_50_ values of usenamine A for H1299 and A549 cells were 2.004 and 2.4 μg/mL, respectively (*p* < 0.05). Usenamine A dramatically reduced the proliferation of H1299 and A549 cells (*p* < 0.01; Figure 1A, 1B and Supplementary Figure 1A). Wound-healing and transwell assays showed that usenamine A could significantly suppress the migrative and invasive abilities of H1299 and A549 cells, respectively (*p* < 0.05; Figure 1C, 1D and Supplementary Figure 1B, 1C). Flow cytometric analysis revealed that usenamine A increased the apoptotic ratio of H1299 and A549 cells (*p* < 0.05; Figure 1E and Supplementary Figure 1D). In addition, we examined the efficacy of usenamine A on pyroptosis in LUAD cells. It has been reported that the amplification of reactive oxygen species (ROS) signals can drive cell pyroptosis [20]. We found that usenamine A treatment increased ROS levels in H1299 and A549 cells (Figure 2A and Supplementary Figure 2A). In addition, pyroptosis contributes to lactate dehydrogenase (LDH) release and promotes inflammation [21]. As shown in Figure 2B and Supplementary Figure 2B, usenamine A treatment significantly enhanced LDH release and levels of proinflammatory cytokine (IL-1β and IL-18) in H1299 and A549 cells (*p* < 0.05). Usenamine A treatment induced notable cytoplasmic swelling and membrane rupture in H1299 and A549 cells (Figure 2C and Supplementary Figure 2C). Caspases are a family of conserved cysteine proteases, regulating pyroptosis and inflammation [22]. Herein, we detected the levels of caspases (caspase-1, -4, -5, and-8) in LUAD cells after usenamine A treatment using RT-qPCR. Based on the RT-qPCR results, usenamine A treatment markedly increased caspase-1 levels in H1299 and A549 cells (*p* < 0.05), whereas levels of caspase-4, -5, and-8 did not exhibit significant changes (Figure 2D and Supplementary Figure 2D). Furthermore, NLRP3/caspase-1/GSDMD pathway has been reported to regulate pyroptosis [23]; hence, we measured pathway-related proteins. Western blotting showed that usenamine A treatment upregulated the expression of NLRP3, ASC, Cleaved-caspase1/pro-caspase1, and GSDMD-N/GSDMD-F in H1299 and A549 cells (*p* < 0.05; Figure 2E and Supplementary Figure 2E). Notably, usenamine A dose-dependently inhibited LUAD cell development (*p* < 0.05).

**Figure 3:**
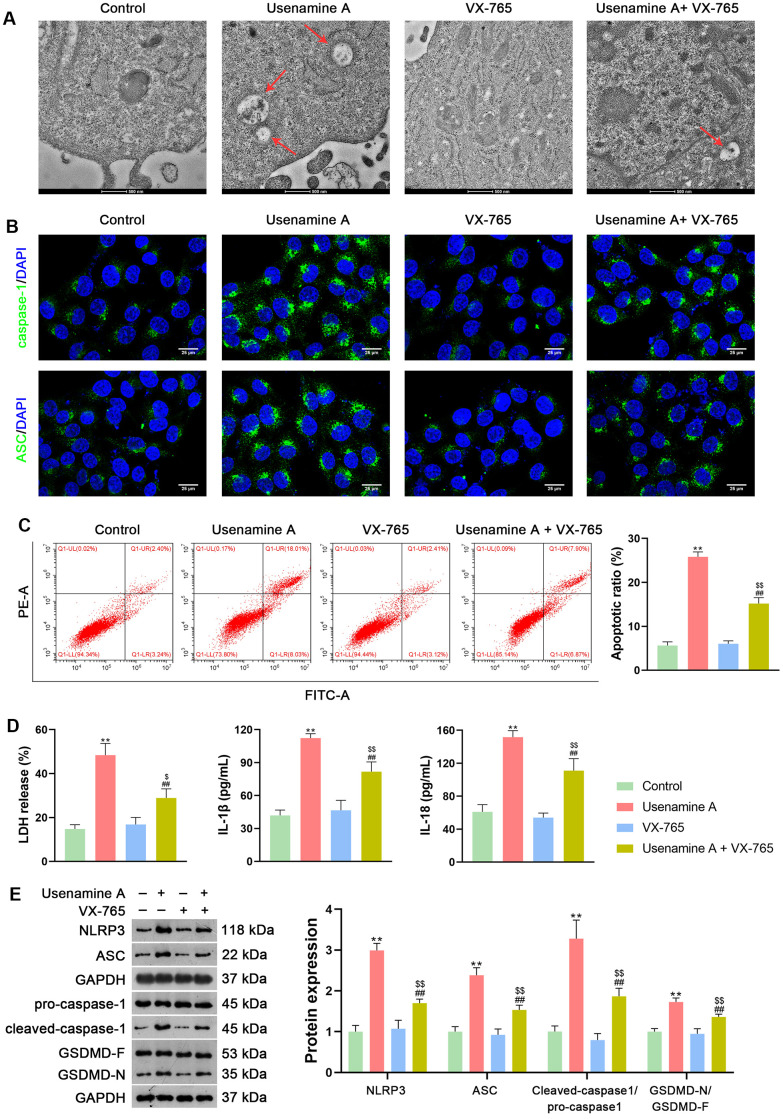
**Usenamine A triggers NLRP3/caspase-1/GSDMD-mediated pyroptosis in LUAD cells (H1299), as determined by complementary validation.** (**A**) Cell pyroptosis was observed using TEM. Scale bar = 500 nm. (**B**) Expression of caspase-1 and ASC was measured using immunofluorescence staining. Scale bar = 25 μm. (**C**) Cell apoptosis was detected by flow cytometry. (**D**) LDH, IL-1β, and IL-18 levels in cells were measured using commercial ELISA kits. (**E**) Relative protein expression of NLRP3/caspase-1/GSDMD pathway-related proteins was measured using western blotting. **p < 0.01 vs*.* Control; ##*p* < 0.01 vs*.* usenamine A (2 μg/mL); $*p* < 0.05 and $$*p* < 0.01 vs*.* VX-765 (20 μM). GSDMD, gasdermin D; LUAD, lung adenocarcinoma; NLRP3, NOD-like receptor pyrin 3.

To further verify the facilitatory effect of usenamine A on LUAD cell pyroptosis, usenamine A-treated H1299 cells were simultaneously treated with 2 μg/mL VX-765. VX-765, a non-toxic caspase-1 inhibitor, suppresses caspase-1-dependent pyroptosis effectively [24]. Transmission electron microscopy (TEM) revealed that usenamine A-treated cells displayed features of pyroptosis, including cell swelling and low cytosol density, whereas the addition of VX-765 reduced these pyroptosis features (Figure 3A). Furthermore, VX-765 co-treatment decreased caspase-1 and ASC levels in usenamine A-treated cells (Figure 3B). VX-765 reduced the apoptotic ratio of usenamine A-treated H1299 cells (*p* < 0.01; Figure 3C). Additionally, the levels of LDH, IL-1β, and IL-18 in usenamine A-treated cells were significantly reduced following the addition of VX-765 treatment (*p* < 0.01; Figure 3D). The expression of NLRP3/caspase-1/GSDMD pathway-related proteins (NLRP3, ASC, Cleaved-caspase1/pro-caspase1, and GSDMD-N/GSDMD-F) was measured and demonstrated significant downregulation in usenamine A-treated cells exposed to VX-765 (*p* < 0.01; Figure 3E).

### Usenamine A triggers pyroptosis of LUAD cells by upregulating DDX3X

**Figure 4:**
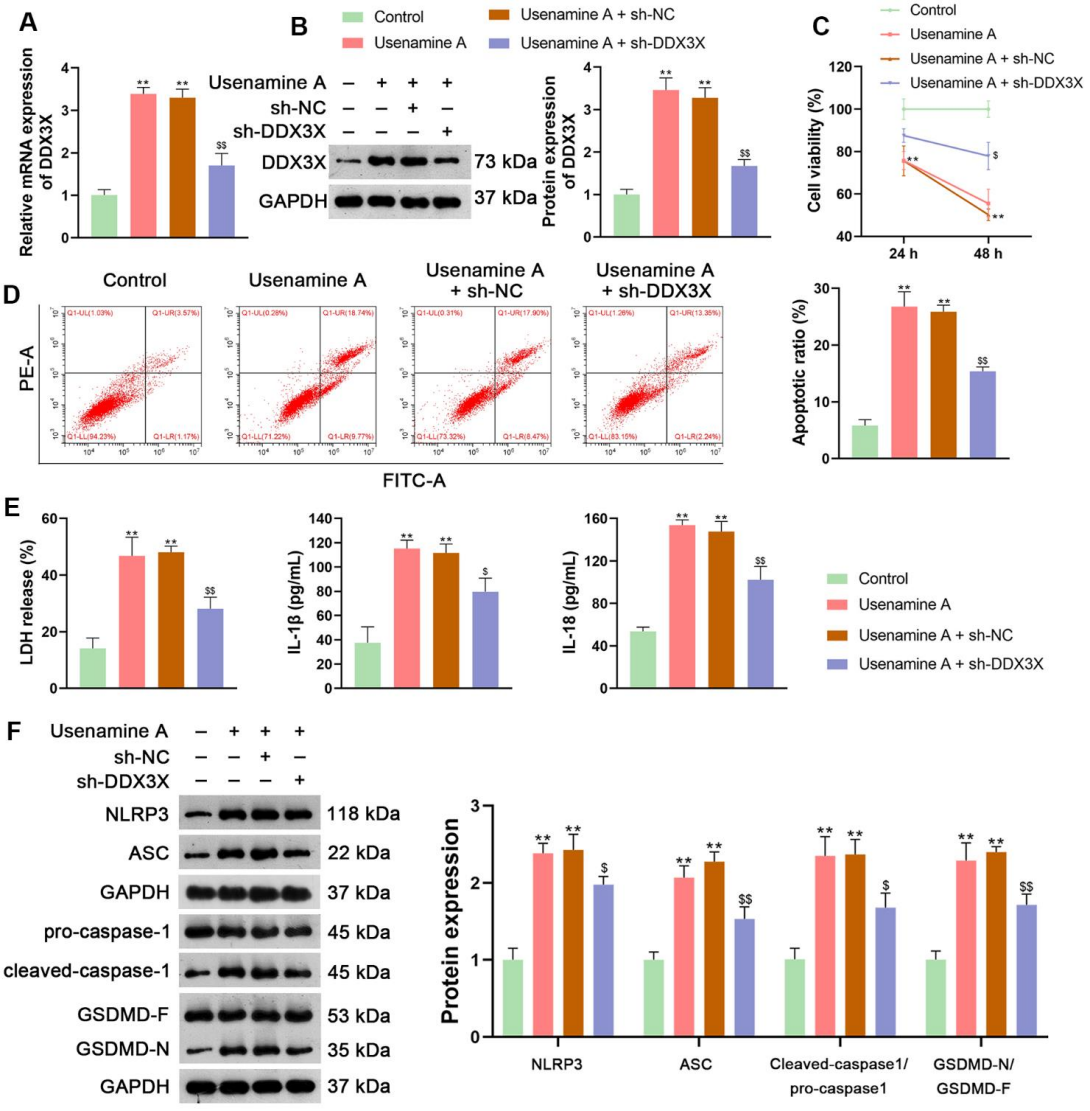
**Usenamine A promotes LUAD cell pyroptosis by upregulating DDX3X.** (**A**, **B**) Relative mRNA and protein expression of DDX3X were measured by RT-qPCR and western blotting, respectively. (**C**) H1299 cell viability was determined using the CCK-8 assay. (**D**) H1299 cell apoptosis was measured using flow cytometry. (**E**) LDH, IL-1β, and IL-18 levels in cells were measured using commercial ELISA kits. (**F**) Relative protein expression of NLRP3/caspase-1/GSDMD pathway-related proteins was measured using western blotting. *p < 0.05 and **p < 0.01 vs*.* Control; $*p* < 0.05 and $$*p* < 0.01 vs*.* usenamine A (2 μg/mL) + sh-NC. DDX3X, DEAD-Box helicase 3 X-Linked; GSDMD, gasdermin D; LUAD, lung adenocarcinoma; NLRP3, NOD-like receptor pyrin 3; RT-qPCR, reverse transcription-quantitative PCR.

DDX3X has been reported to regulate NLRP3 inflammasome-induced pyroptosis [25]. To explore whether usenamine A could induce pyroptosis in LUAD cells via DDX3X, H1299 cells treated with usenamine A were then transfected with sh-DDX3X. Figure 4A, 4B shows that usenamine A upregulated the expression of DDX3X in H1299 cells, while sh-DDX3X transfection significantly knocked down DDX3X expression (*p* < 0.01). DDX3X knockdown enhanced the viability and reduced the apoptosis of usenamine A-treated H1299 cells (*p* < 0.01; Figure 4C, 4D). Additionally, DDX3X knockdown lowered LDH, IL-1β, and IL-18 levels in usenamine A-treated cells (*p* < 0.05; Figure 4E). Following DDX3X knockdown, there was downregulation of proteins related to the NLRP3/caspase-1/GSDMD pathway in usenamine A-treated cells (*p* < 0.05; Figure 4F).

### DDX3X promotes NLRP3/caspase-1/GSDMD-mediated pyroptosis in LUAD cells by interacting with SQSTM1

**Figure 5:**
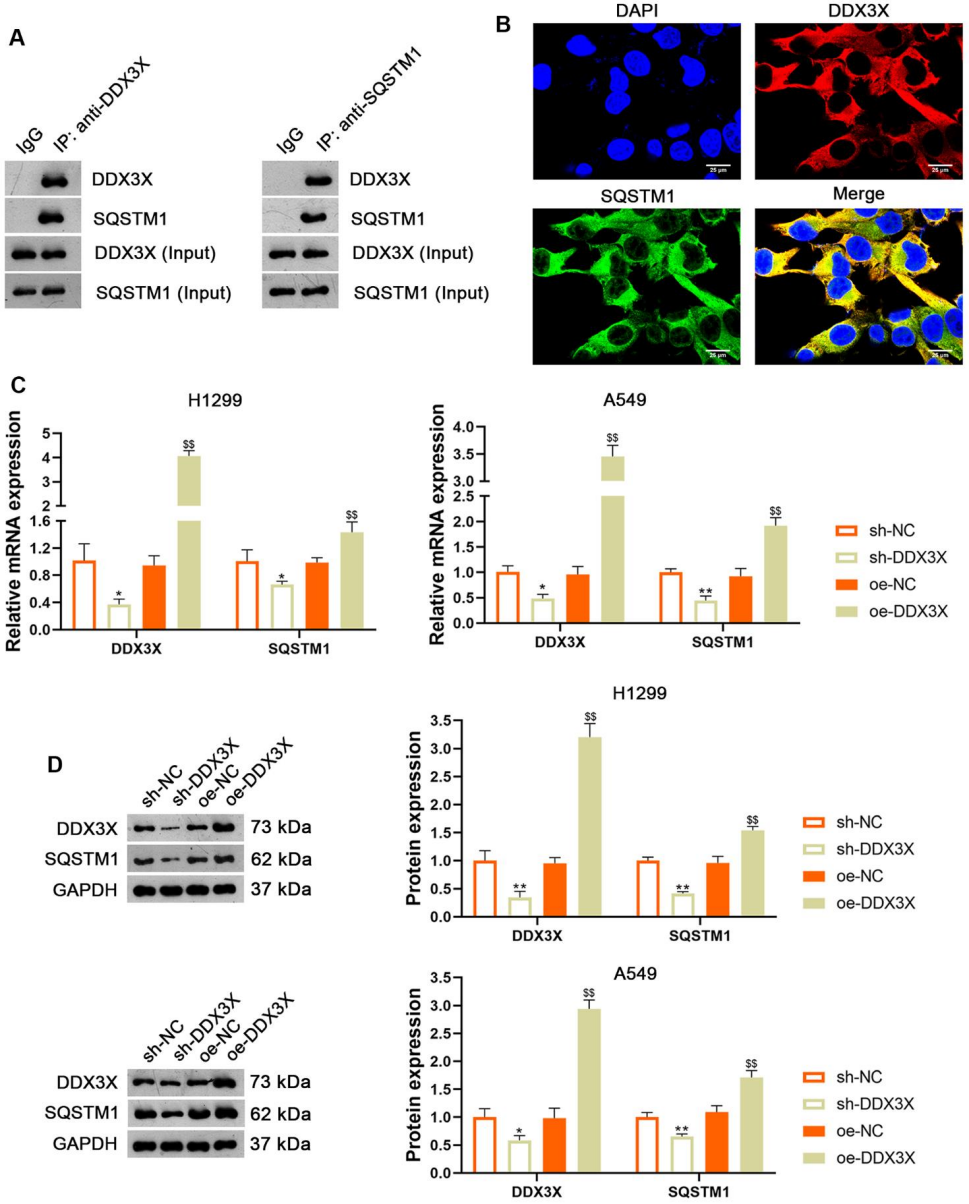
**DDX3X interacts with SQSTM1 in LUAD cells.** (**A**) The interaction between DDX3X and SQSTM1 was identified by co-immunoprecipitation. (**B**) Immunofluorescence detected the subcellular co-location of DDX3X and SQSTM1. Scale bar = 25 μm. (**C**, **D**) Relative mRNA and protein expression of DDX3X and SQSTM1 in H1299 and A549 cells were measured by RT-qPCR and western blotting, respectively. *p < 0.05 and **p < 0.01 vs*.* sh-NC; $$*p* < 0.01 vs*.* oe-NC. DDX3X, DEAD-Box helicase 3 X-Linked; SQSTMI1, sequestosome 1; LUAD, lung adenocarcinoma; RT-qPCR, reverse transcription-quantitative PCR.

**Figure 6:**
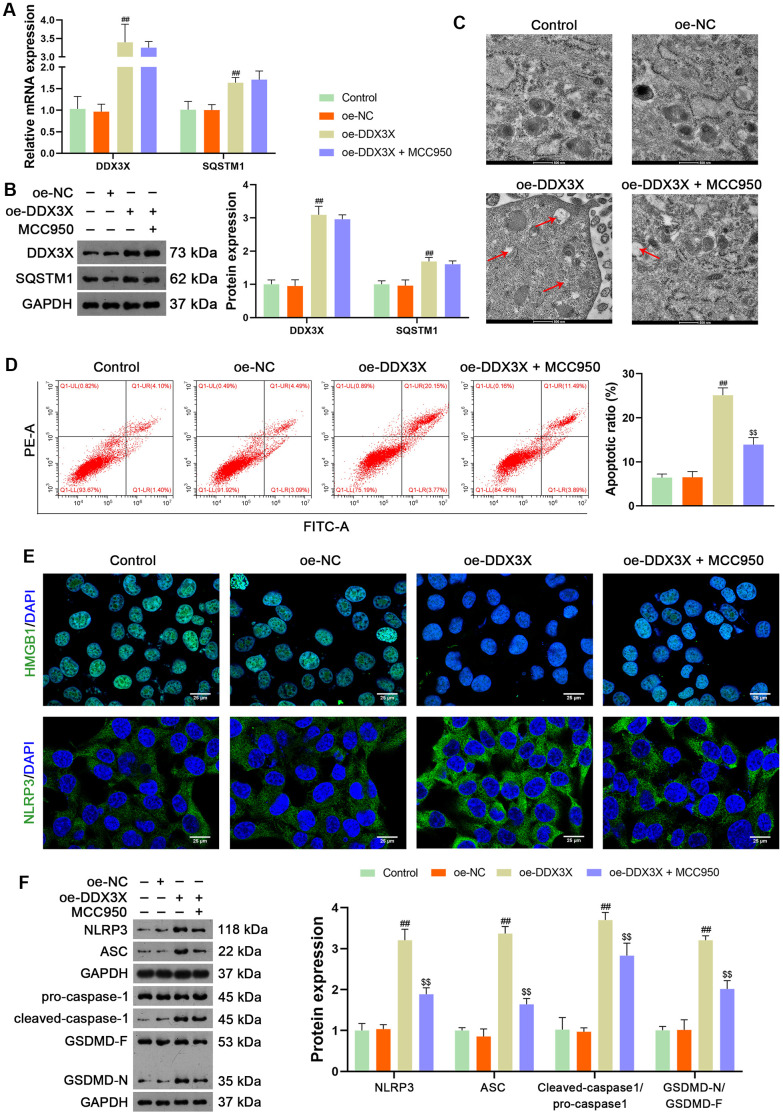
**DDX3X overexpression promotes NLRP3/caspase-1/GSDMD-mediated pyroptosis in LUAD cells.** (**A**, **B**) Relative mRNA and protein expression of DDX3X and SQSTM1 in H1299 cells were measured by RT-qPCR and western blotting, respectively. (**C**) Cell pyroptosis was observed by TEM. Scale bar = 500 nm. (**D**) H1299 cell apoptosis was measured by flow cytometry. (**E**) Expression of HMGB1 and NLRP3 were measured by immunofluorescence staining. Scale bar = 25 μm. (**F**) Relative protein expression of NLRP3/caspase-1/GSDMD pathway-related proteins was measured by western blotting. ##*p* < 0.01 vs*.* oe-NC; $$*p* < 0.01 vs*.* oe-DDX3X. DDX3X, DEAD-Box helicase 3 X-Linked; GSDMD, gasdermin D; LUAD, lung adenocarcinoma; NLRP3, NOD-like receptor pyrin 3; RT-qPCR, reverse transcription-quantitative PCR.

DDX3X reportedly exhibits oncogenic activity by facilitating p62 accumulation [16]. The Co-immunoprecipitation (Co-IP) assay verified the interaction between DDX3X and SQSTM1 (Figure 5A). Immunofluorescence staining showed that DDX3X and SQSTM1 co-localized in the cytoplasm (Figure 5B). To confirm the regulatory effect of DDX3X on SQSTM1 in LUAD cells, DDX3X was knocked down or overexpressed in H1299 and A549 cells. Results showed that DDX3X knockdown led to a decrease in SQSTM1 expression in both H1299 and A549 cells, while DDX3X overexpression increased SQSTM1 levels (*p* < 0.05; Figure 5C, 5D). To assess if DDX3X promotes NLRP3/caspase-1/GSDMD-mediated pyroptosis, DDX3X-overexpressed H1299 cells were treated with MCC950, an NLRP3 inflammasome inhibitor. MCC950 suppresses pyroptosis by inhibiting NLRP3 inflammasome activation and the subsequent caspase-1-mediated cleavage of GSDMD [26]. Figure 6A, 6B shows that the addition of MCC950 did not significantly impact the expression of DDX3X and SQSTM1 in H1299 cells. In functional terms, H1299 cells with overexpressed DDX3X showed clear signs of pyroptosis, which were mitigated by the addition of MCC950 (Figure 6C). In addition, flow cytometry revealed that DDX3X overexpression promoted apoptosis in H1299 cells, which was reduced following MCC950 addition (*p* < 0.05; Figure 6D). Immunofluorescence staining revealed that DDX3X overexpression led to the downregulation of HMGB1 and the upregulation of NLRP3 in H1299 cells (Figure 6E). Western blotting revealed that DDX3X overexpression increased levels of NLRP3, ASC, Cleaved-caspase1/pro-caspase1, and GSDMD-N/GSDMD-F (*p* < 0.01; Figure 6F). Notably, the addition of MCC950 could weaken the facilitatory effect of DDX3X on the NLRP3/caspase-1/GSDMD pathway (*p* < 0.01; Figure 6E, 6F).

### Usenamine A inhibits the tumor growth of LUAD by promoting DDX3X/SQSTM1 axis *in vivo*

**Figure 7:**
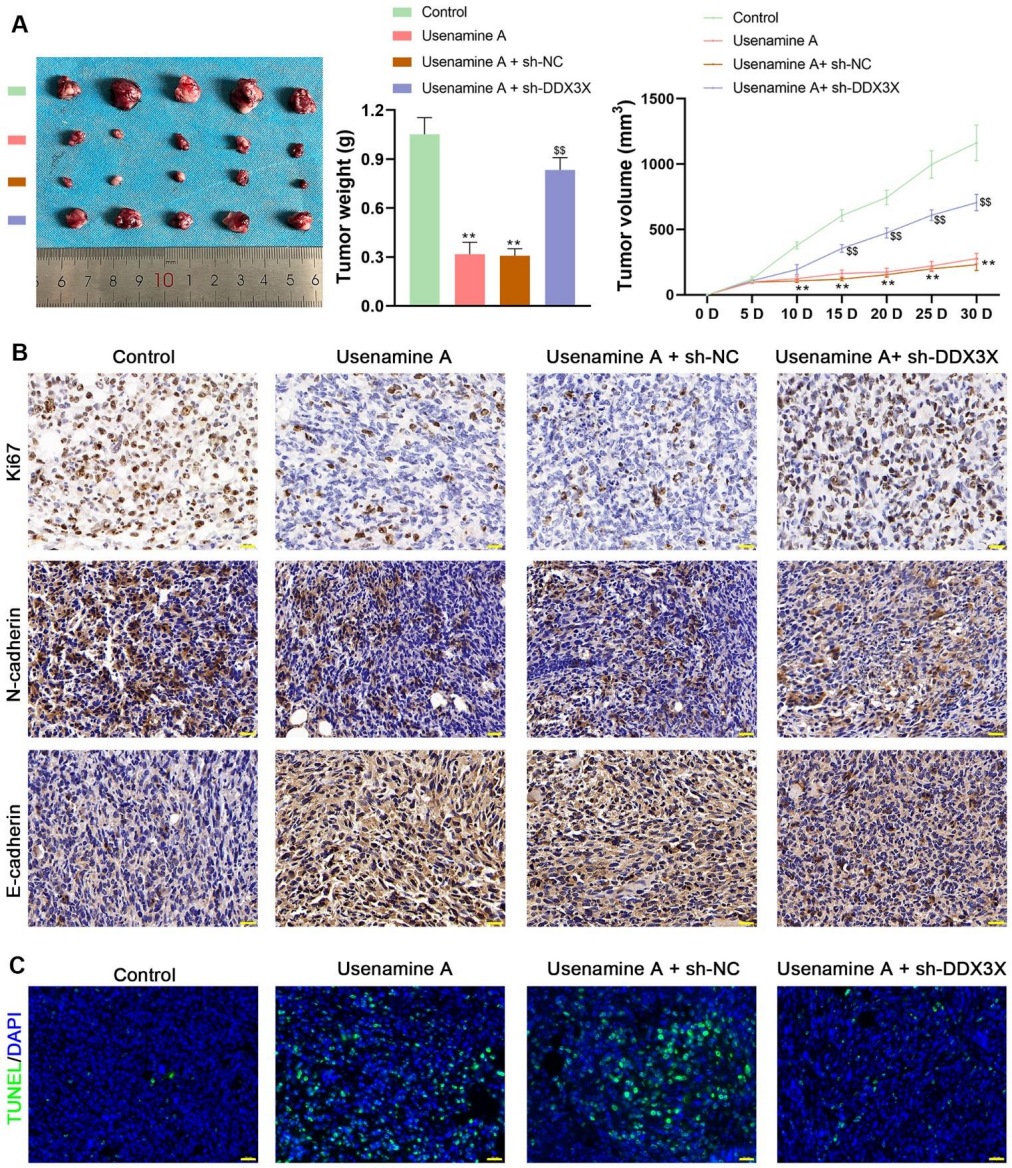
**Usenamine A inhibits LUAD tumor growth by regulating DDX3X.** promotes NLRP3/caspase-1/GSDMD-mediated pyroptosis by upregulating the DDX3X/SQSTM1 axis *in vivo*. (**A**) Tumor weight and volume. **p < 0.01 vs*.* control; $$*p* < 0.01 vs*.* usenamine A + sh-DDX3X. (**B**) Protein expression of Ki67, N-cadherin, and E-cadherin in tumor tissues was measured by immunohistochemical staining. Scale bar = 20 μm. (**C**) Apoptosis in tissues was detected by TUNEL staining. Scale bar = 20 μm. LUAD, lung adenocarcinoma; DDX3X, DEAD-Box helicase 3 X-Linked.

**Figure 8:**
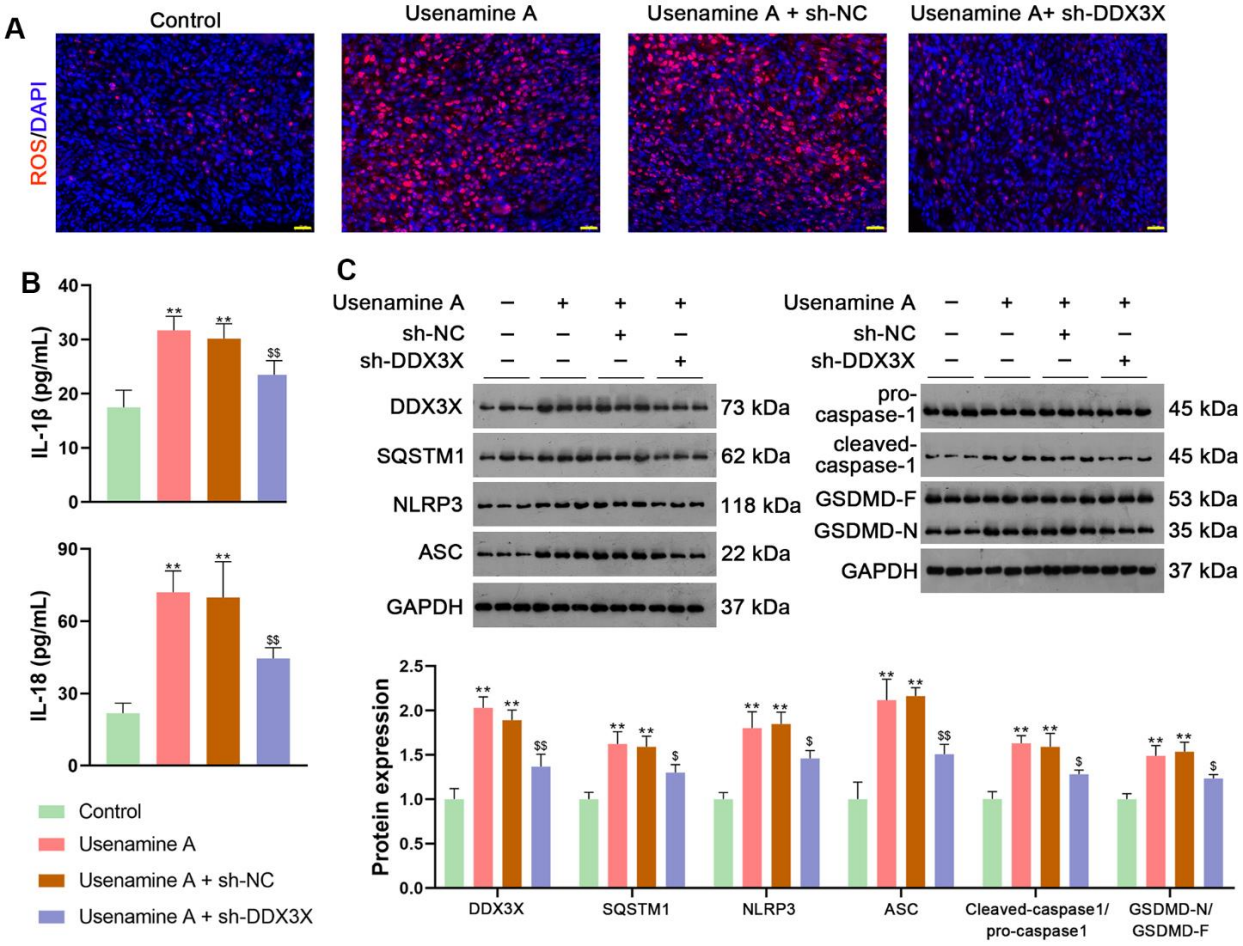
**Usenamine A promotes NLRP3/caspase-1/GSDMD-mediated pyroptosis by upregulating the DDX3X/SQSTM1 axis *in vivo*.** (**A**) ROS levels in the tissues were determined using a DCFH-DA probe. Scale bar = 20 μm. (**B**) Serum levels of IL-1β and IL-18 were measured using enzyme-linked immunosorbent assay kits. (**C**) Relative expression of DDX3X, SQSTM1, and NLRP3/caspase-1/GSDMD pathway-related proteins was monitored using western blotting. **p < 0.01 vs*.* control; $*p* < 0.05 and $$*p* < 0.01 vs*.* usenamine A + sh-DDX3X. DDX3X, DEAD-Box helicase 3 X-Linked; GSDMD, gasdermin D; LUAD, lung adenocarcinoma; NLRP3, NOD-like receptor pyrin 3; RT-qPCR, reverse transcription-quantitative PCR; SQSTMI1, sequestosome 1.

BALB/c mice treated with usenamine A or sh-DDX3X were used to confirm the effects and mechanisms of usenamine A on LUAD *in vivo*. In usenamine A-treated mice, tumor weight and volume were significantly suppressed (*p* < 0.01; Figure 7A). Moreover, immunohistochemistry (IHC) staining revealed that usenamine A treatment downregulated the expression of Ki67 and N-cadherin but upregulated E-cadherin expression (Figure 7B). TUNEL staining results showed that usenamine A promoted apoptosis in tumor tissues (Figure 7C). Additionally, we observed that usenamine A increased ROS levels in tumor tissues and elevated levels of IL-1β and IL-18 in serum (*p* < 0.01; Figure 8A, 8B). The transfection of sh-DDX3X alleviated the inhibitory effect of usenamine A on LUAD (*p* < 0.01; Figures 7A–7C, 8A, 8B). Finally, usenamine A treatment decreased the expression of DDX3X, SQSTM1, NLRP3, ASC, Cleaved-caspase1/pro-caspase1, and GSDMD-N/GSDMD-F, which was suppressed by sh-DDX3X addition (*p* < 0.05; Figure 8C).

## DISCUSSION

In the current study, we uncovered the inhibitory effect and mechanism of usenamine A on LUAD, as evidenced by the following discoveries: (1) usenamine A repressed the proliferation, migration, and invasion of LUAD cells (H1299 and A549) (Figure 1 and Supplementary Figure 1); (2) usenamine A promoted NLRP3/caspase-1/GSDMD-mediated pyroptosis of LUAD cells (Figure 2 and Supplementary Figure 2); (3) usenamine A upregulated DDX3X to trigger pyroptosis (Figure 4); (4) DDX3X interacts with SQSTM1 (Figure 5), which contributed to the induction of pyroptosis (Figure 6); (5) *In vivo* experiments validated that usenamine A suppressed LUAD tumorigenesis by triggering NLRP3/caspase-1/GSDMD-mediated pyroptosis, which was achieved by upregulating DDX3X/ SQSTM1 axis (Figure 7).

Usenamine A (C_18_H_17_NO_6_) is a novel natural anticancer drug with favorable antitumor effects and low toxicity in multiple cancers (Patent No.: 201710388136.8). The combination of usenamine A and scutellarin inhibits glioma cell proliferation and migration and promotes apoptosis [10, 11]. Yang et al. demonstrated that usenamine A induces apoptosis and autophagic cell death in human hepatoma cells [27]. Usenamine A was found to effectively inhibit cell proliferation in lung cancer, with an IC_50_ value of 1.68 μM (Patent No.: 201710388136.8). Consistently, we demonstrated that usenamine A could suppress LUAD cell proliferation, migration, and invasion, and promote apoptosis, with an IC_50_ value of 2 μg/mL. Pyroptosis is inflammatory programmed cell death mediated by the NLRP3/caspase-1/GSDMD pathway [28]. Accumulating research has focused on NLRP3/caspase-1/GSDMD-mediated pyroptosis to explore novel effective anticancer drugs and their molecular mechanisms [21, 29, 30]. Yang et al. have revealed that hydrogen exerts an antitumor effect on endometrial cancer via the NLRP3/caspase-1/GSDMD-mediated pyroptotic pathway [28]. The natural product triptolide, which exerts potent antitumor activity, has been confirmed to induce GSDME-mediated pyroptosis in head and neck cancer [31]. Yuan et al. have reported that cucurbitacin B triggers NLRP3/GSDMD-dependent pyroptosis to retard non-small cell lung cancer [32]. Herein, we found that usenamine A increased inflammatory levels and enhanced NLRP3/caspase-1/GSDMD-mediated pyroptosis in LUAD cells. Moreover, the addition of VX-765 (a caspase-1 inhibitor) reversed the stimulatory effect of usenamine A on LRP3/caspase-1/GSDMD-mediated pyroptosis. These findings indicate that usenamine A inhibits LUAD by inducing NLRP3/caspase-1/GSDMD-mediated pyroptosis.

Furthermore, the mechanism through which usenamine A promotes pyroptosis in LUAD was investigated. DDX3X is a member of the Asp-Glu-Ala-Asp (DEAD)-box helicase family, involved in apoptosis, cell cycle, cellular stress response, and innate immunity in multiple diseases, including cancer [33, 34]. DDX3X critically regulates the activation of the NLRP3 inflammasome, leading to pyroptosis [34, 35]. Our results demonstrated that usenamine A promoted the expression of DDX3X in LUAD cells. DDX3X knockdown suppressed the effect of usenamine A on NLRP3/caspase-1/GSDMD-mediated pyroptosis. These results suggest that usenamine A triggers NLRP3/caspase-1/GSDMD-mediated pyroptosis by upregulating DDX3X expression in LUAD cells.

Reportedly, DDX3X induces epithelial-mesenchymal transition in pancreatic ductal adenocarcinoma by promoting SQSTM1 accumulation [16], indicating the interaction between DDX3X and SQSTM1. In the present study, Co-IP and immunofluorescence assays confirmed the interaction and co-localization of DDX3X and SQSTM1 in the cytoplasm. SQSTM1 is a ubiquitin-binding autophagy receptor that exhibits oncogenic activity in various cancers [16, 36]. Moreover, SQSTM1 accumulation can exacerbate NLRP3- and GSDMD-mediated pyroptosis [18]. Therefore, we speculate that usenamine A induces NLRP3/caspase-1/GSDMD-mediated pyroptosis in LUAD by targeting the DDX3X/SQSTM1 axis. Our results showed that DDX3X overexpression upregulated SQSTM1 expression and promoted NLRP3/caspase-1/GSDMD-mediated pyroptosis in LUAD cells. In addition, MCC950, an inhibitor of NLRP3 inflammasome, reversed the stimulatory effects of oe-DDX3X on SQSTM1 expression and pyroptosis. These findings confirm the essential positive role of DDX3X/SQSTM1 in mediating the effects of usenamine A, triggering pyroptosis in LUAD. Finally, *in vivo* experiments validated that usenamine A impedes LUAD tumorigenesis and induces pyroptosis by upregulating the DDX3X/SQSTM1 axis.

This study examines the anti-cancer effects of usenamine A in LUAD and identifies the underlying mechanism involving the activation of NLRP3/caspase-1/GSDMD-mediated pyroptosis. However, there are some potential limitations and confounding variables. Firstly, further validation with additional LUAD cell lines and primary human tissue samples will strengthen the credibility of these findings. Second, the specificity of DDX3X-SQSTM1 binding needs to be explored to rule out any alternative contributors to pyroptosis regulation in LUAD cells. Lastly, the *in vivo* study was restricted to a single strain and dosing regimen, necessitating further investigations to account for inter-strain, gender-based, and dosage-specific differences. Despite these caveats, the findings provide encouraging evidence supporting the continued development of usenamine A as a treatment option for LUAD patients.

## CONCLUSIONS

**Figure 9:**
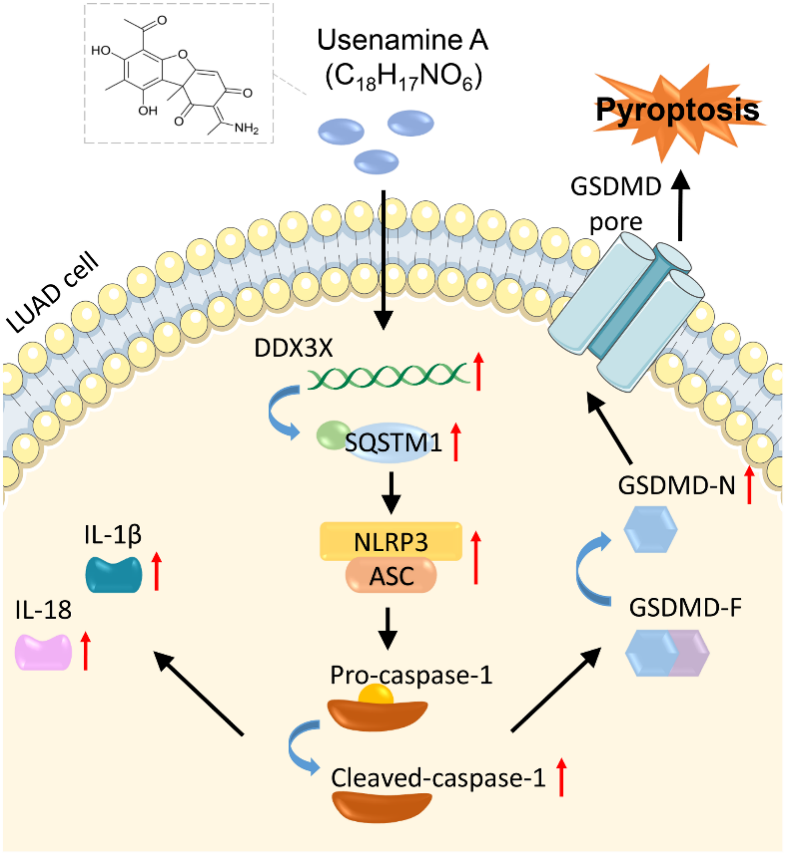
Mechanisms of usenamine A inducing pyroptosis in LUAD by targeting the DDX3X/SQSTM1 axis and activating the NLRP3/caspase-1/GSDMD pathway.

In summary, usenamine A suppresses LUAD progression by inducing NLRP3/caspase-1/GSDMD-mediated pyroptosis. This process is achieved by upregulating the DDX3X/SQSTM1 axis. Our study revealed a promising auxiliary therapeutic drug for LUAD. Moreover, we elucidated the mechanism through which usenamine A induces pyroptosis in LUAD, specifically by targeting the DDX3X/SQSTM1 axis and activating the NLRP3/caspase-1/GSDMD pathway (Figure 9). Our study lays a strong foundation for developing novel therapeutic approaches for LUAD. We plan to expand upon our findings by conducting investigations on other LUAD cell lines and primary patient tissues, clarifying the mechanism regulating DDX3X/SQSTM1.

## MATERIALS AND METHODS

### Cell culture and treatment

The normal human lung epithelial cell line (BEAS-2B), LUAD cell lines (H1299 and A549), and HEK-293T cells were obtained from the FuHeng Cell Center (Shanghai, China). All cells were maintained in Dulbecco’s modified Eagle’s medium (Gibco, Grand Island, NY, USA) supplemented with 10% fetal bovine serum (FBS;Thermo Fisher Scientific, MA, USA) and 1% penicillin/streptomycin (Hyclone, MA, USA). All cells were cultured in a humidified atmosphere containing 5% CO_2_ at 37° C. All treatments were performed on cells at passage three to ensure uniformity.

Cell treatment 1: H1299 and A549 cells were treated with gradient concentrations of usenamine A (0, 1, 2, and 3 μg/mL) for 24 h. The concentrations of usenamine A used in our study were selected based on Patent No.: 201710388136.8 and a previous study [37]. Morphology of cells was observed using a microscope (Olympus, Japan). Usenamine A was provided by Xiaoqiong He, School of Public Health, Kunming Medical University.

Treatment 2: H1299 cells were administrated with 2 μg/mL usenamine A and/or 20 μM VX-765 (a caspase-1 inhibitor; HY-13205, MCE, China) for 24 h.

Treatment 3: The short hairpin RNAs (shRNAs) of negative control (NC) and DDX3X (sense sequence:5′-GGA GTT CTA GCA AAG ATA A-3′, antisense sequence:5′-TTA TCT TTG CTA GAA CTC C-3′) were obtained by GenePharma Co. Ltd. (Shanghai, China). Sh-DDX3X or sh-NC were transfected into H1299 cells by lentivirus packaging using HighGene transfection reagent (ABclonal, MA, USA) for 72 h. After transfection, cells were administrated with 2 μg/mL usenamine A for 24 h.

Treatment 4: H1299 and A549 cells were transfected with sh-NC, sh-DDX3X, overexpression (oe-NC, or oe-DDX3X by lentivirus packaging using HighGene transfection reagent (ABclonal) for 72 h. Oe-DDX3X H1299 cells were treated with 10 μM MCC950 (an NLRP3 inflammasome inhibitor; HY-12815, MCE) for 8 h.

The successful transfection of H1299 cells with sh-DDX3X, oe-DDX3X or NC was confirmed by RT-qPCR and Western blot analysis, ensuring the effectiveness of gene silencing or overexpression.

### CCK-8 assay

The viability of H1299 and A549 cells was evaluated using the CCK-8 assay. Briefly, H1299 and A549 cells were seeded in 96-well plates (100 μL per well) at 37° C at an atmosphere of 5% CO_2_. After treatment at 24 and 48 h, CCK-8 solution (10 μL) was added into each well and incubated for 2 h. Cell viability was measured using a microplate reader (Wuxi Hiwell-Diatek, China) at 450 nm.

### Colony formation assay

Cells were resuspended and then plated into a six-well plate (200 cells per well) to obtain a 14-day culture in a Heracell™ 150i CO_2_ incubator (Thermo Fisher Scientific, MA, USA). Next, cells were fixed in methanol (1 mL per well) for 15 min and then stained with crystal violet (Beyotime, China) for 20 min. Excess staining solution was washed with phosphate-buffered saline (PBS), and cells were captured by a digital camera (Olympus, Japan).

### Wound-healing assay

Cell suspension was seeded into six-well plates (5 × 10^5^ cells per well). At the second day, a pipette tip (10 μL) was applied to create equal-width linear scratches on the six-well plates. Cell fragments were washed with PBS, and cell images were captured at 0, 12, and 24 h under a DMI3000 B inverted fluorescence phase contrast microscope (Leica, Germany). Wound-healing rate (%) = average distance at (0 h–x h)/0 h × 100.

### Transwell assay

At 48 h following treatment, cells were digested with trypsin and resuspended in serum-free medium to 1 × 10^5^/mL. For the migration assay, the upper chamber of the Transwell (24-well insert, 8 μm pores, Corning, NY, USA) was filled with 200 μL cell suspension, and the lower chamber with 10% FBS medium. Considering the invasion assay, the upper chamber was covered with Matrigel (BD Biosciences). After 48-h incubation, cells in the lower chamber were rinsed twice with PBS and then immobilized with methanol for 30 min. Next, cells were stained with crystal violet for 20 min and photographed using a fluorescence microscope (Olympus, Japan) in three random fields.

### Flow cytometry

Apoptosis was examined using an Annexin V-FITC Apoptosis Detection Kit (Beyotime, China). Cells were digested with 0.25% trypsin and centrifuged for 5 min at 1,500 rpm to collect cells. After washing twice with PBS, 300 μL of binding buffer was used to resuspend cells. The cell suspension was incubated with annexin V-FITC (5 μL) for 15 min and with 10 μL propidium iodide for 10 min under dark conditions. Apoptotic cells were detected using a CytoFLEX S Flow Cytometer (Beckman Coulter, CA, USA) and analyzed using the CellQuest Pro software.

### RT-qPCR

Total RNA was extracted from cells using TRIzol reagent (Invitrogen, CA, USA). Complementary DNA was obtained using the FastKing-RT SuperMix (Tiangen, China). RT-qPCR was carried out using SYBR Green PCR Master Mix (Lifeint, China) with the Mx3000P Multiplex Quantitative PCR (QPCR) System (Agilent Stratagene, CA, USA). The thermocycling conditions were 95° C for 3 min, followed by 40 cycles of 95° C for 12 s and 62° C for 40 s. The relative gene expression was obtained by the 2^-ΔΔCT^ method. Primer sequences used are presented in Table 1.

**Table 1:** Primer sequences for RT-qPCR.

**Gene**	**Primer sequences (5’-3’)**	**Size (bp)**
Caspase-1 (human)	Forward: ACAAGACCTCTGACAGCACG	489 bp
	Reverse: GCATCTGCGCTCTACCATCT	
Caspase-4 (human)	Forward: AGGGCATTTGCTACCAGACC	166 bp
	Reverse: GGCAGTTGCGGTTGTTGAAT	
Caspase-5 (human)	Forward: GTGCCCAGAGTTGAAGGAGTC	411 bp
	Reverse: TTGATGAGCCACGCGATTCT	
Caspase-8 (human)	Forward: GCTGACTTTCTGCTGGGGAT	112 bp
	Reverse: GACATCGCTCTCTCAGGCTC	
DDX3X (human)	Forward: ACCAACGAGAGAGTTGGCAG	946 bp
	Reverse: AGAACGTCCACGACTGCTAC	
SQSTM1 (human)	Forward: CATCGGAGGATCCGAGTGTG	475 bp
	Reverse: AGATGTGGGTACAAGGCAGC	
GAPDH (human)	Forward: TGTGGGCATCAATGGATTTGG	116 bp
	Reverse: ACACCATGTATTCCGGGTCAAT	

### TEM

Cells were fixed with 2.5% glutaraldehyde, dehydrated with ethanol and acetone, embedded, and cut into slices. Next, sections were stained with 2% uranyl acetate and lead citrate. Finally, cells were observed under a Tecnai F30 transmission electron microscope (FEI, CA, USA) at a voltage of 80 kV.

### Immunofluorescence staining

The cells were plated into 12-well plates and fixed with 3% formaldehyde (Sinopharm, China) for 15 min. After washing three times with PBS, the cells were permeabilized with Triton-X 100 (1%) for 10 min. Subsequently, cells were blocked with bovine serum albumin (3%) for 30 min. Next, cells were labeled with primary antibodies including anti-caspase-1 (1:500; AF5418, Affinity, USA), anti-ASC (1:100; DF6304, Affinity), anti-DDX3X (1:50; ab271002, Abcam, UK), anti-SQSTM1 (1 μg/mL; ab109012, Abcam), anti-HMGB1 (1 μg/mL; ab18256, Abcam), and anti-NLRP3 (1:100; ab263899, Abcam) overnight at 4° C. Following that, cells were incubated with the secondary antibody FITC goat anti-rabbit IgG (H+L) (ABclonal, MA, USA) and 4’,6-diamidino-2-phenylindole (DAPI, 1:500) for 1 h in the dark. Finally, the cells were visualized using an UltraVIEW VoX spinning disk confocal microscope (PerkinElmer, MA, USA).

### Co-IP assay

To determine the interactions between DDX3X and SQSTM1, HEK-293T cells were lysed with IP buffer, and supernatant was obtained by centrifuging for 1 min at 1,000 ×*g*. The supernatant was incubated with beads carrying DDX3X and SQSTM1 antibodies for 12 h. Then, beads were washed with lysis buffer and mixed with sodium dodecyl sulfate (SDS) buffer for western blot analysis.

### Tumor xenografts animal experiments

Animal study was approved by the Institutional Animal Care and Use Committee of Xiamen University (XMULAC20220034-9). Specific pathogen-free 6–8 weeks old BALB/c mice (n = 20) were obtained from GemPharmatech Co., Ltd. (Nanjing, China, animal license #: SCXK-2018–0027). All mice were acclimatized under a 12-h light/dark cycle at 22 ± 1° C. After one week, non-transfected or sh-NC/sh-DDX3X-transfected H1299 cells (3 × 10^6^ cells per mouse) were administered subcutaneously to mice. Tumor size was monitored every five days. Tumor volume measurements were determined using the formula V (mm^3^) = (width)^2^ × length/2. When the tumor volume reached 180-200 mm^3^, the mice were subcutaneously injected with 2 mg/kg usenamine A every three days. Control mice were injected with 0.9% normal saline. After four weeks or upon reaching humane endpoints, which were rigorously defined by clinical signs such as severe lethargy, inability to obtain food or water, significant weight loss, or tumor burden exceeding ethical limits, animals were euthanized using CO_2_ inhalation—a method aligned with the latest AVMA guidelines for humane euthanasia. The euthanasia process involved gradual filling of the chamber with CO_2_ to ensure minimal distress. Tumors were then collected for further analysis.

### IHC

Tumor tissues were fixed with 10% formaldehyde for 48 h, dehydrated with ethanol, and then embedded in paraffin for cutting into 4 μm sections. Sections were then deparaffinized with xylene, rehydrated, and blocked with goat serum for 15 min. Next, sections were incubated with primary antibodies against Ki67 (5 μg/mL; ab15580, Abcam, UK), N-cadherin (1 μg/mL; ab18203, Abcam), and E-cadherin (1:80,000; ab76319, Abcam) overnight at 4° C. Following that, sections were incubated with a secondary antibody (1:2,000; ab6721, Abcam) for 15 min. Sections were then visualized using 3,3′-diaminobenzidine (DAB; Beyotime, China) solution and stained with hematoxylin for 3 min. Tissue images were photographed by a microscope (Olympus, Tokyo, Japan).

### TUNEL staining

Apoptosis in tumor tissues was detected using the One-Step TUNEL Apoptosis Assay Kit (Beyotime, China). Briefly, tumor tissue sections were digested using proteinase K solution at 37° C for 30 min. After washing with PBS three times, samples were stained with TUNEL reagent and then counterstained with DAPI for 1 h at 37° C under dark conditions. TUNEL staining was captured using a fluorescence microscope (Olympus, Tokyo, Japan).

### ROS detection

Cell suspensions and tumor tissue sections were incubated with DCFH-DA reagent (10 μM; Solarbio, China) for 40 min and DAPI staining solution for 15 min at 37° C. Subsequently, the cells were photographed using a fluorescence microscope and analyzed using Image J software (National Institutes of Health, Bethesda, MD).

### Enzyme-linked immunosorbent assay (ELISA)

Cells were plated in 96-well plates (8 × 10^3^ cells per well) for 24 h. LDH release from cells was measured by the CyQUANT LDH Cytotoxicity Assay kit (Thermo Fisher Scientific, MA, USA). Levels of IL-1β and IL-18 in LUAD cells and serum were measured using IL-1β and IL-18 ELISA kits (Mlbio, China), respectively.

### Western blot assay

Cells and tumor tissues were lysed by radioimmunoprecipitation assay (RIPA) buffer (Beyotime, China) with phenylmethanesulfonyl fluoride (PMSF) protease inhibitor (Beyotime) to extract total protein. Proteins (25 μg per well) were separated using 10% SDS-PAGE and transferred onto polyvinylidene fluoride (PVDF) membranes (Beyotime). Membranes were blocked with 5% skim milk for 1 h, and then incubated with primary antibodies overnight at 4° C. After washing with 1× TBST containing 0.1% Tween-20, membranes were incubated with a secondary antibody (1:2,000; ab6721, Abcam) in the dark for 1 h. Protein bands were visualized by the ECL chemiluminescence solution (APPLYGEN, China). The primary antibodies used for western blotting are presented in Supplementary Table 1.

### Statistical analyses

Animal experiments were conducted with quintuplicate samples (n = 5), and cell-based assays were performed in triplicate (n = 3). Data values are expressed as mean ± standard deviation. Differences between pairs of groups were assessed with an unpaired two-tailed Student’s t-test. For analyses involving more than two groups, we employed one-way ANOVA. Subsequent to ANOVA, we conducted post-hoc pairwise comparisons applying Tukey’s Honest Significant Difference (HSD) test, which corrects for the multiple comparison problem. A *P*-value of < 0.05 was considered significant.

## Supplementary Material

Supplementary Figures

Supplementary Table 1
