# Supplementary material for: Usenamine A triggers NLRP3/caspase-1/GSDMD-mediated pyroptosis in lung adenocarcinoma by targeting the DDX3X/SQSTM1 axis
Source: Aging (Albany NY). 2024 Jan 23;16(2):1663–84. doi: 10.18632/aging.205450 (PMC10866397; doi:10.18632/aging.205450)
Supplement: Supplementary Figures [file aging-16-205450-s001.pdf]

SUPPLEMENTARY FIGURES

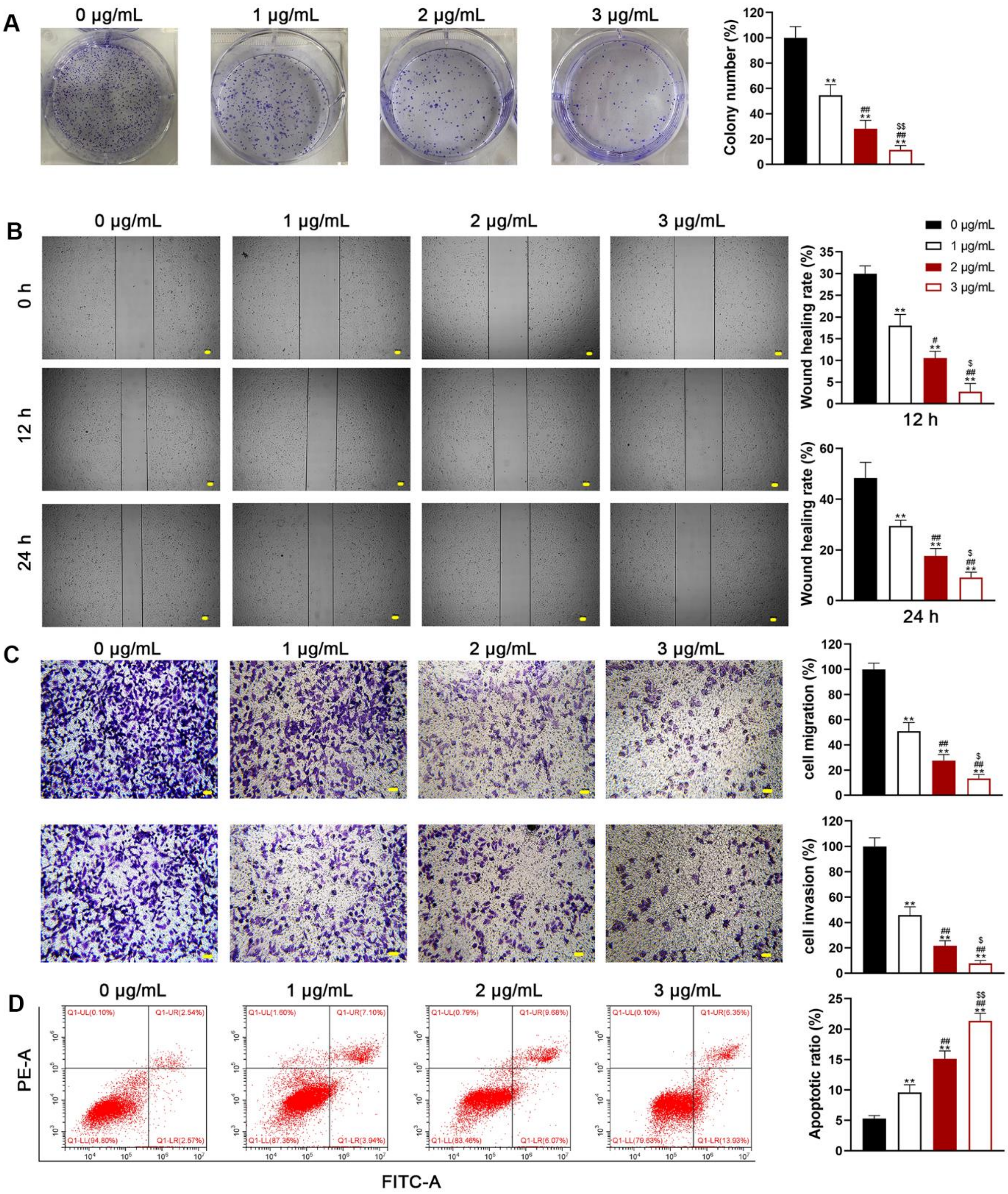

Supplementary Figure 1. Usenamine A inhibits the proliferation, migration, and invasion of LUAD cells (A549). (**A**) Cell proliferation was detected using a colony formation assay. (**B**) Cell migration was measured using a wound-healing assay. Scale bar = 50 μm. (**C**) Cell migration and invasion were determined using the transwell assay. Scale bar = 50 μm. (**D**) Cell apoptosis was assessed using flow cytometry. $^{**}p < 0.01$ *vs.* 0 μg/mL usenamine A; $\#p < 0.05$ and $\#\#p < 0.01$ *vs.* 1 μg/mL usenamine A; $\$p < 0.05$ and $\$\$p < 0.01$ *vs.* 2 μg/mL usenamine A.

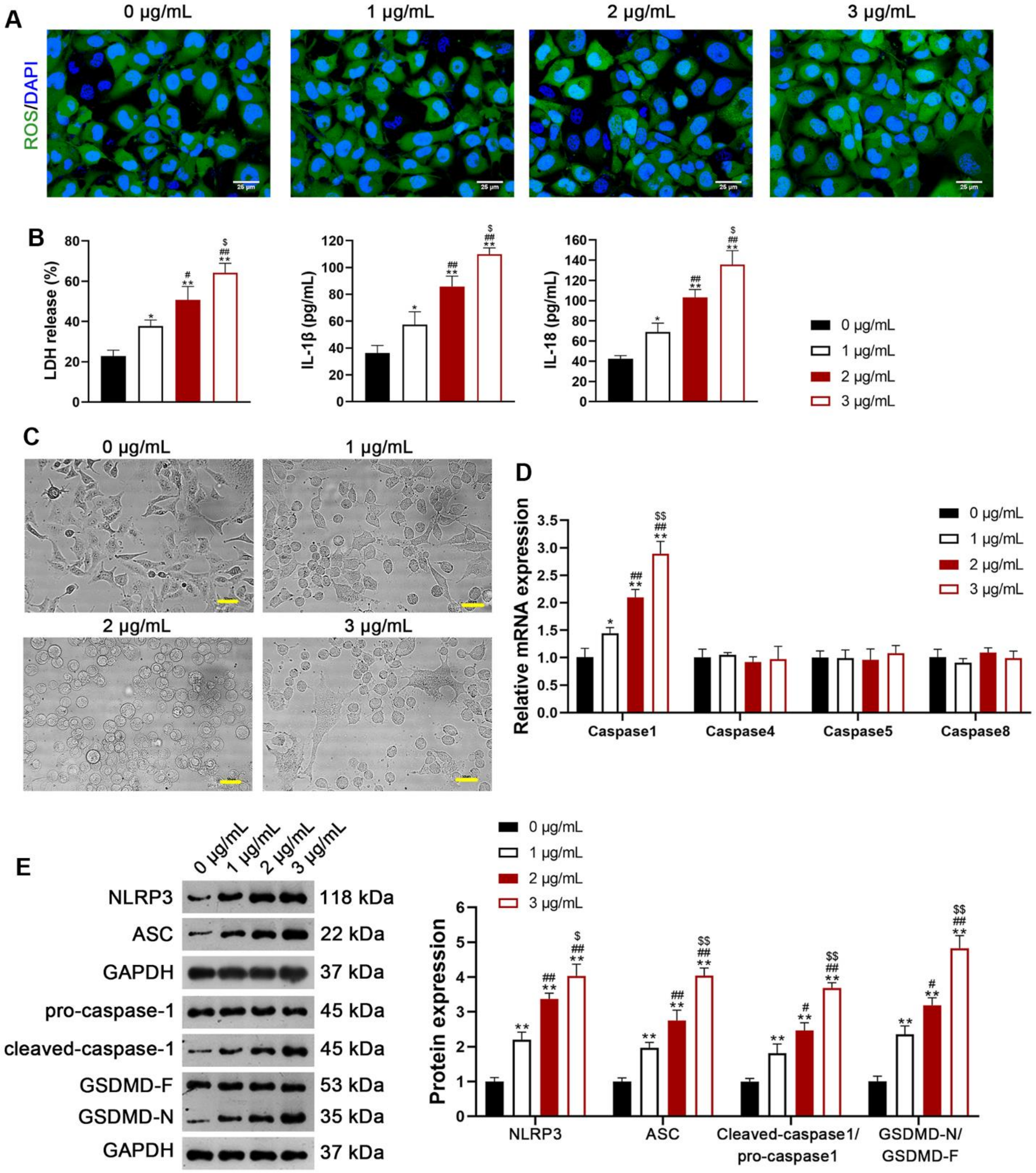

Supplementary Figure 2. Usenamine A induces NLRP3/caspase-1/GSDMD-mediated pyroptosis in LUAD cells (A549). (**A**) Levels of ROS in the cells were detected using a DCFH-DA probe. Scale bar = 25 μm. (**B**) LDH, IL-1β, and IL-18 levels in the cells were measured using commercial ELISA kits. (**C**) Representative morphological images of cells obtained by microscopic examination. Scale bar = 50 μm. (**D**) Relative expression of caspase-1, -4, -5, and -8 was analyzed using RT-qPCR. (**E**) Relative protein expression of NLRP3/caspase-1/GSDMD pathway-related proteins was measured using western blotting. *$p < 0.05$ and **$p < 0.01$ *vs.* 0 μg/mL usenamine A; #$p < 0.05$ and ##$p < 0.01$ *vs.* 1 μg/mL usenamine A; \$$p < 0.05$ and \$\$$p < 0.01$ *vs.* 2 μg/mL usenamine A. GSDMD, gasdermin D; LUAD, lung adenocarcinoma; NLRP3, NOD-like receptor pyrin 3; RT-qPCR, reverse transcription-quantitative PCR.
