# Supplementary material for: Usenamine A triggers NLRP3/caspase-1/GSDMD-mediated pyroptosis in lung adenocarcinoma by targeting the DDX3X/SQSTM1 axis
Source: Aging (Albany NY). 2024 Jan 23;16(2):1663–84. doi: 10.18632/aging.205450 (PMC10866397; doi:10.18632/aging.205450)
Supplement: Supplementary Table 1 [file aging-16-205450-s002.pdf]

SUPPLEMENTARY TABLE

Supplementary Table 1. Primary antibodies for western blotting.

Antibodies	Catalog number	Supplier	Working concentration
Anti-NLRP3	ab263899	Abcam, UK	1:1,000
Anti-ASC	DF6304	Affinity, CA, USA	1:1,000
Cleaved Caspase 1	AF5418	Affinity, CA, USA	1:1,000
Anti-pro Caspase-1	ab179515	Abcam, UK	1:1,000
GSDMD N-Terminal	DF13758	Affinity, CA, USA	1:500
GSDMD F	AF4012	Affinity, CA, USA	1:1,000
DDX3X	ab271002	Abcam, UK	1:1,000
Anti-SQSTM1	ab109012	Abcam, UK	1:10,000
Anti-GAPDH	ab245355	Abcam, UK	1:10,000
